# *N*,*N*′-Bis(2-methoxy­phen­yl)biphenyl-2,2′-dicarboxamide

**DOI:** 10.1107/S1600536808032352

**Published:** 2008-10-11

**Authors:** Gui-Yu Wang, Di Li, Da-Bin Qin, Jie-Wei Luo, Li-Hui Guo

**Affiliations:** a School of Chemistry and Chemical Engineering, China West Normal University, Nanchong 637002, People’s Republic of China

## Abstract

In the title compound, C_28_H_24_N_2_O_4_, the dihedral angle between the two rings of the biphenyl unit is 75.34 (9)°. The outer aromatic rings form dihedral angles of 66.96 (1) and 85.69 (8)° with the rings to which they are attached . The mol­ecular structure is stabilized by intra­molecular C—H⋯O and N—H⋯O hydrogen bonds. In the crystal structure, inter­molecular N—H⋯O inter­actions are observed.

## Related literature

For the synthesis, see: Gao & Gao (2002 ▶). For related structures, see: Wang & Han (2004 ▶); Wang & Jiang (2004 ▶); Huang & Yang (2008 ▶).
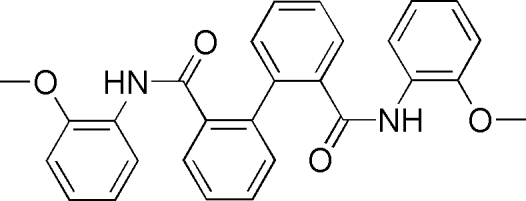


## Experimental

### 

#### Crystal data

C_28_H_24_N_2_O_4_
*M* *_r_* = 452.49
Monoclinic, 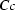

*a* = 18.184 (4) Å
*b* = 16.304 (3) Å
*c* = 7.9998 (16) Å
β = 108.90 (3)°
*V* = 2243.9 (8) Å^3^
*Z* = 4
Mo *K*α radiation
μ = 0.09 mm^−1^
*T* = 113 (2) K
0.16 × 0.14 × 0.10 mm

#### Data collection

Rigaku Saturn CCD diffractometer
Absorption correction: multi-scan (*CrystalStructure*; Rigaku/MSC, 2004 ▶) *T*_min_ = 0.986, *T*_max_ = 0.991
6422 measured reflections
1991 independent reflections
1858 reflections with *I* > 2σ(*I*)
*R*_int_ = 0.054

#### Refinement

*R*[*F* ^2^ > 2σ(*F* ^2^)] = 0.039
*wR*(*F* ^2^) = 0.093
*S* = 1.06
1991 reflections
309 parameters
2 restraints
H-atom parameters constrained
Δρ_max_ = 0.17 e Å^−3^
Δρ_min_ = −0.20 e Å^−3^

### 

Data collection: *RAPID-AUTO* (Rigaku/MSC, 2004 ▶); cell refinement: *RAPID-AUTO*; data reduction: *RAPID-AUTO*; program(s) used to solve structure: *SHELXS97* (Sheldrick, 2008 ▶); program(s) used to refine structure: *SHELXL97* (Sheldrick, 2008 ▶); molecular graphics: *SHELXTL* (Sheldrick, 2008 ▶); software used to prepare material for publication: *SHELXTL*.

## Supplementary Material

Crystal structure: contains datablocks global, I. DOI: 10.1107/S1600536808032352/gw2044sup1.cif

Structure factors: contains datablocks I. DOI: 10.1107/S1600536808032352/gw2044Isup2.hkl

Additional supplementary materials:  crystallographic information; 3D view; checkCIF report

## Figures and Tables

**Table 1:** Hydrogen-bond geometry (Å, °)

*D*—H⋯*A*	*D*—H	H⋯*A*	*D*⋯*A*	*D*—H⋯*A*
N1—H1N⋯O3^i^	0.86	2.02	2.833 (3)	157
N2—H2N⋯O2	0.86	2.24	3.081 (4)	167
N2—H2N⋯O4	0.86	2.24	2.612 (3)	106
C22—H22⋯O3	0.93	2.30	2.885 (4)	120

Symmetry code: (i) 
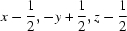
.

**Fig. 1.**
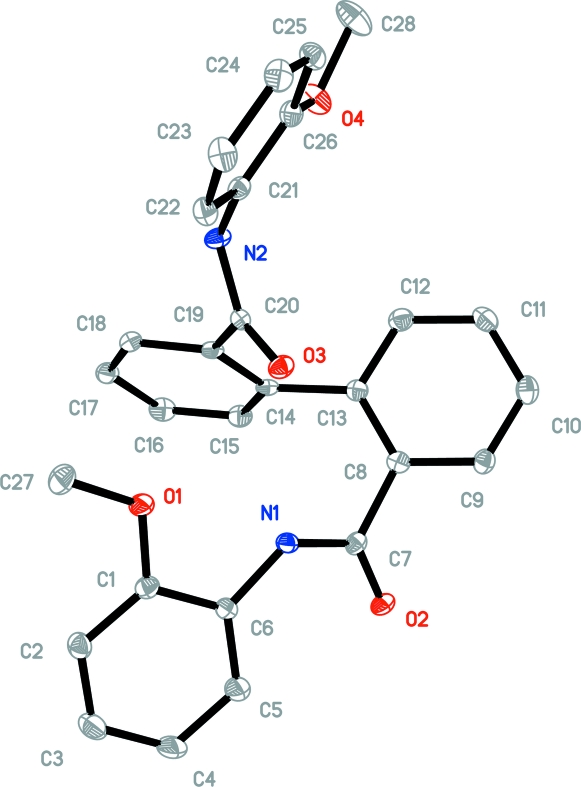
The molecular structure of the title compound, showing 30% probability displacement ellipsoids and the atomic numbering.

## supplementary crystallographic information

### Comment

The distortion of diphenyl spacer about central bond not only endows dpa a peculiar characterization to link metal ions or metal clusters into macrocycles or helical chains, but also makes diphenic acid (H2dpa) can deprotonate partially forming hydrogen bonds of carboxylic groups to meet both geometric and energetic requirements. We here report the crystal structure of the title compound.

The C8—C13 and C14—C19 planes form the dihedral angle of 75.34 (9)°, and C1—C6 ring are nearly perpendicular to C14—C19 ring, with a dihedral angle of 85.69 (8)°.The molecular structure is stabilized by C—H···O and N—H···O intramolecular hydrogen bonds.In addition, weak C—H···O intermolecular hydrogen bonds are observed.

### Experimental

The title compound was prepared according to the reported procedure of *M. Z*. Gao & Gao (2002). Colourless single crystals suitable for X-ray diffraction were obtained by recrystallization from dimethyl sulfoxide.

### Refinement

H atoms were placed in calculated positions with C—H = 0.93 Å, and N—H = 0.86 Å,and refined in riding mode with *U*_iso_(H) = 1.2*U*_eq_(C,*N*).

### Crystal data

**Table:**

C_28_H_24_N_2_O_4_	*F*(000) = 952
*M**_r_* = 452.49	*D*_x_ = 1.339 Mg m^−^^3^
Monoclinic, *C**c*	Mo *K*α radiation, λ = 0.71073 Å
*a* = 18.184 (4) Å	Cell parameters from 3190 reflections
*b* = 16.304 (3) Å	θ = 1.7–27.9°
*c* = 7.9998 (16) Å	µ = 0.09 mm^−^^1^
β = 108.90 (3)°	*T* = 113 K
*V* = 2243.9 (8) Å^3^	Block, colourless
*Z* = 4	0.16 × 0.14 × 0.10 mm

### Data collection

**Table:**

Rigaku Saturn diffractometer	1991 independent reflections
Radiation source: rotating anode	1858 reflections with *I* > 2σ(*I*)
confocal	*R*_int_ = 0.054
ω scans	θ_max_ = 25.0°, θ_min_ = 1.7°
Absorption correction: multi-scan (CrystalStructure; Rigaku/MSC, 2004)	*h* = −21→20
*T*_min_ = 0.986, *T*_max_ = 0.991	*k* = −17→19
6422 measured reflections	*l* = −9→9

### Refinement

**Table:**

Refinement on *F*^2^	Primary atom site location: structure-invariant direct methods
Least-squares matrix: full	Secondary atom site location: difference Fourier map
*R*[*F*^2^ > 2σ(*F*^2^)] = 0.039	Hydrogen site location: inferred from neighbouring sites
*wR*(*F*^2^) = 0.093	H-atom parameters constrained
*S* = 1.06	*w* = 1/[σ^2^(*F*_o_^2^) + (0.0537*P*)^2^] where *P* = (*F*_o_^2^ + 2*F*_c_^2^)/3
1991 reflections	(Δ/σ)_max_ = 0.007
309 parameters	Δρ_max_ = 0.17 e Å^−^^3^
2 restraints	Δρ_min_ = −0.20 e Å^−^^3^

### Special details

**Table:**

Geometry. All e.s.d.'s (except the e.s.d. in the dihedral angle between two l.s. planes) are estimated using the full covariance matrix. The cell e.s.d.'s are taken into account individually in the estimation of e.s.d.'s in distances, angles and torsion angles; correlations between e.s.d.'s in cell parameters are only used when they are defined by crystal symmetry. An approximate (isotropic) treatment of cell e.s.d.'s is used for estimating e.s.d.'s involving l.s. planes.
Refinement. Refinement of *F*^2^ against ALL reflections. The weighted *R*-factor *wR* and goodness of fit *S* are based on *F*^2^, conventional *R*-factors *R* are based on *F*, with *F* set to zero for negative *F*^2^. The threshold expression of *F*^2^ > σ(*F*^2^) is used only for calculating *R*-factors(gt) *etc*. and is not relevant to the choice of reflections for refinement. *R*-factors based on *F*^2^ are statistically about twice as large as those based on *F*, and *R*- factors based on ALL data will be even larger.

### Fractional atomic coordinates and isotropic or equivalent isotropic displacement parameters (Å^2^)

**Table:**

	*x*	*y*	*z*	*U*_iso_*/*U*_eq_	
O1	−0.01240 (15)	0.26417 (14)	0.9137 (3)	0.0338 (6)	
O2	0.21854 (13)	0.17729 (12)	1.0081 (2)	0.0234 (5)	
O3	0.45976 (12)	0.32159 (13)	1.0239 (2)	0.0239 (5)	
O4	0.31213 (13)	0.07137 (13)	0.8380 (3)	0.0273 (5)	
N1	0.09211 (15)	0.17216 (15)	0.8317 (3)	0.0222 (6)	
H1N	0.0615	0.1801	0.7258	0.027*	
N2	0.37809 (15)	0.21050 (14)	0.9645 (3)	0.0216 (6)	
H2N	0.3350	0.1932	0.9743	0.026*	
C1	0.00360 (19)	0.19053 (18)	0.9988 (4)	0.0229 (7)	
C2	−0.0328 (2)	0.16090 (19)	1.1154 (4)	0.0277 (7)	
H2	−0.0694	0.1928	1.1441	0.033*	
C3	−0.0139 (2)	0.0831 (2)	1.1889 (4)	0.0298 (8)	
H3	−0.0378	0.0632	1.2674	0.036*	
C4	0.0399 (2)	0.0355 (2)	1.1457 (4)	0.0305 (8)	
H4	0.0512	−0.0170	1.1926	0.037*	
C5	0.07701 (19)	0.06580 (18)	1.0327 (3)	0.0258 (7)	
H5	0.1148	0.0345	1.0072	0.031*	
C6	0.05817 (18)	0.14248 (18)	0.9576 (3)	0.0210 (7)	
C7	0.16777 (18)	0.18877 (17)	0.8643 (3)	0.0188 (6)	
C8	0.18756 (18)	0.22120 (18)	0.7085 (3)	0.0201 (7)	
C9	0.16253 (18)	0.17982 (18)	0.5469 (4)	0.0219 (7)	
H9	0.1304	0.1342	0.5336	0.026*	
C10	0.18522 (19)	0.20637 (19)	0.4066 (3)	0.0251 (7)	
H10	0.1695	0.1779	0.3000	0.030*	
C11	0.2317 (2)	0.27591 (19)	0.4260 (4)	0.0275 (7)	
H11	0.2471	0.2940	0.3321	0.033*	
C12	0.25501 (19)	0.31821 (19)	0.5843 (4)	0.0252 (7)	
H12	0.2853	0.3651	0.5956	0.030*	
C13	0.23333 (17)	0.29096 (18)	0.7281 (3)	0.0190 (6)	
C14	0.25474 (18)	0.34128 (17)	0.8950 (3)	0.0204 (6)	
C15	0.1978 (2)	0.39160 (18)	0.9223 (4)	0.0270 (7)	
H15	0.1475	0.3901	0.8428	0.032*	
C16	0.2152 (2)	0.4441 (2)	1.0670 (4)	0.0307 (8)	
H16	0.1763	0.4766	1.0847	0.037*	
C17	0.2897 (2)	0.44812 (19)	1.1841 (4)	0.0290 (8)	
H17	0.3015	0.4837	1.2800	0.035*	
C18	0.34668 (19)	0.39879 (18)	1.1576 (4)	0.0244 (7)	
H18	0.3972	0.4021	1.2355	0.029*	
C19	0.32979 (19)	0.34399 (18)	1.0156 (3)	0.0211 (6)	
C20	0.39544 (18)	0.29130 (17)	1.0011 (3)	0.0210 (7)	
C21	0.42452 (18)	0.15199 (18)	0.9115 (3)	0.0212 (7)	
C22	0.5013 (2)	0.1627 (2)	0.9232 (4)	0.0317 (8)	
H22	0.5276	0.2099	0.9756	0.038*	
C23	0.5398 (2)	0.1035 (2)	0.8573 (5)	0.0407 (9)	
H23	0.5918	0.1111	0.8674	0.049*	
C24	0.5015 (2)	0.0338 (2)	0.7773 (4)	0.0354 (8)	
H24	0.5271	−0.0048	0.7305	0.042*	
C25	0.4250 (2)	0.0217 (2)	0.7669 (4)	0.0275 (8)	
H25	0.3988	−0.0252	0.7127	0.033*	
C26	0.38714 (18)	0.07931 (18)	0.8372 (3)	0.0225 (7)	
C28	0.2734 (2)	−0.0051 (2)	0.7829 (5)	0.0366 (9)	
H28A	0.2719	−0.0168	0.6642	0.055*	
H28B	0.2214	−0.0019	0.7873	0.055*	
H28C	0.3011	−0.0480	0.8601	0.055*	
C27	−0.0779 (3)	0.3085 (2)	0.9290 (5)	0.0458 (10)	
H27A	−0.1234	0.2746	0.8892	0.069*	
H27B	−0.0856	0.3571	0.8578	0.069*	
H27C	−0.0684	0.3234	1.0502	0.069*	

### Atomic displacement parameters (Å^2^)

**Table:**

	*U*^11^	*U*^22^	*U*^33^	*U*^12^	*U*^13^	*U*^23^
O1	0.0436 (16)	0.0284 (13)	0.0359 (11)	0.0087 (11)	0.0218 (11)	0.0078 (9)
O2	0.0200 (12)	0.0303 (12)	0.0181 (10)	−0.0008 (9)	0.0038 (9)	0.0020 (8)
O3	0.0182 (13)	0.0273 (12)	0.0252 (10)	−0.0060 (9)	0.0054 (10)	−0.0002 (8)
O4	0.0194 (12)	0.0223 (12)	0.0408 (11)	−0.0041 (9)	0.0104 (10)	−0.0049 (9)
N1	0.0164 (14)	0.0333 (15)	0.0163 (11)	−0.0017 (11)	0.0045 (11)	0.0036 (9)
N2	0.0172 (14)	0.0205 (14)	0.0279 (13)	0.0000 (10)	0.0084 (11)	−0.0017 (10)
C1	0.0267 (18)	0.0191 (15)	0.0228 (14)	−0.0017 (13)	0.0076 (13)	0.0001 (11)
C2	0.030 (2)	0.0306 (19)	0.0255 (14)	0.0003 (14)	0.0135 (15)	−0.0028 (12)
C3	0.039 (2)	0.0303 (19)	0.0251 (14)	−0.0089 (15)	0.0176 (16)	0.0009 (12)
C4	0.042 (2)	0.0234 (18)	0.0272 (16)	0.0002 (14)	0.0133 (16)	0.0033 (12)
C5	0.030 (2)	0.0248 (17)	0.0225 (14)	0.0023 (14)	0.0082 (14)	−0.0017 (12)
C6	0.0203 (17)	0.0250 (17)	0.0163 (12)	−0.0035 (12)	0.0042 (13)	−0.0008 (11)
C7	0.0183 (17)	0.0183 (15)	0.0201 (14)	0.0011 (12)	0.0066 (14)	−0.0023 (11)
C8	0.0134 (16)	0.0294 (18)	0.0170 (12)	0.0040 (12)	0.0045 (12)	0.0016 (11)
C9	0.0174 (17)	0.0245 (17)	0.0219 (14)	−0.0004 (13)	0.0036 (13)	−0.0011 (11)
C10	0.0213 (19)	0.0347 (18)	0.0182 (13)	0.0025 (14)	0.0050 (13)	−0.0018 (11)
C11	0.028 (2)	0.0348 (19)	0.0219 (14)	0.0019 (14)	0.0117 (14)	0.0081 (12)
C12	0.0236 (19)	0.0249 (17)	0.0278 (15)	−0.0002 (13)	0.0094 (14)	0.0035 (12)
C13	0.0123 (16)	0.0232 (16)	0.0209 (13)	0.0025 (11)	0.0045 (12)	0.0027 (11)
C14	0.0205 (17)	0.0175 (15)	0.0238 (14)	−0.0011 (12)	0.0079 (13)	0.0020 (11)
C15	0.0231 (18)	0.0254 (17)	0.0305 (15)	0.0006 (14)	0.0057 (14)	0.0010 (12)
C16	0.032 (2)	0.0278 (19)	0.0365 (17)	0.0041 (14)	0.0167 (17)	−0.0022 (13)
C17	0.037 (2)	0.0222 (17)	0.0277 (15)	−0.0017 (14)	0.0105 (15)	−0.0026 (12)
C18	0.0267 (19)	0.0206 (17)	0.0239 (14)	−0.0020 (13)	0.0054 (13)	−0.0010 (11)
C19	0.0221 (17)	0.0192 (16)	0.0234 (14)	0.0007 (12)	0.0091 (14)	0.0030 (11)
C20	0.0223 (19)	0.0250 (17)	0.0143 (13)	−0.0018 (13)	0.0041 (13)	0.0012 (10)
C21	0.0207 (18)	0.0212 (16)	0.0222 (14)	0.0009 (12)	0.0076 (14)	−0.0005 (11)
C22	0.0246 (19)	0.0289 (18)	0.0423 (17)	−0.0024 (14)	0.0118 (16)	−0.0068 (14)
C23	0.0207 (19)	0.042 (2)	0.063 (2)	−0.0016 (15)	0.0182 (18)	−0.0110 (17)
C24	0.034 (2)	0.029 (2)	0.0465 (19)	0.0069 (15)	0.0177 (17)	−0.0012 (14)
C25	0.031 (2)	0.0201 (17)	0.0321 (16)	−0.0004 (13)	0.0110 (15)	−0.0015 (12)
C26	0.0204 (19)	0.0219 (16)	0.0241 (14)	−0.0011 (12)	0.0059 (13)	0.0019 (11)
C28	0.033 (2)	0.031 (2)	0.0477 (19)	−0.0132 (15)	0.0152 (17)	−0.0088 (14)
C27	0.062 (3)	0.037 (2)	0.049 (2)	0.0233 (19)	0.032 (2)	0.0113 (16)

### Geometric parameters (Å, °)

**Table:**

O1—C1	1.364 (4)	C12—C13	1.403 (4)
O1—C27	1.431 (4)	C12—H12	0.9300
O2—C7	1.234 (3)	C13—C14	1.507 (4)
O3—C20	1.228 (4)	C14—C19	1.394 (4)
O4—C26	1.372 (4)	C14—C15	1.393 (4)
O4—C28	1.429 (4)	C15—C16	1.390 (4)
N1—C7	1.342 (4)	C15—H15	0.9300
N1—C6	1.425 (4)	C16—C17	1.377 (5)
N1—H1N	0.8600	C16—H16	0.9300
N2—C20	1.364 (4)	C17—C18	1.381 (5)
N2—C21	1.426 (4)	C17—H17	0.9300
N2—H2N	0.8600	C18—C19	1.398 (4)
C1—C6	1.385 (5)	C18—H18	0.9300
C1—C2	1.393 (4)	C19—C20	1.506 (4)
C2—C3	1.393 (5)	C21—C22	1.380 (5)
C2—H2	0.9300	C21—C26	1.399 (4)
C3—C4	1.377 (5)	C22—C23	1.393 (5)
C3—H3	0.9300	C22—H22	0.9300
C4—C5	1.383 (5)	C23—C24	1.377 (5)
C4—H4	0.9300	C23—H23	0.9300
C5—C6	1.381 (4)	C24—C25	1.381 (5)
C5—H5	0.9300	C24—H24	0.9300
C7—C8	1.501 (4)	C25—C26	1.387 (4)
C8—C13	1.388 (4)	C25—H25	0.9300
C8—C9	1.397 (4)	C28—H28A	0.9600
C9—C10	1.385 (4)	C28—H28B	0.9600
C9—H9	0.9300	C28—H28C	0.9600
C10—C11	1.392 (5)	C27—H27A	0.9600
C10—H10	0.9300	C27—H27B	0.9600
C11—C12	1.383 (4)	C27—H27C	0.9600
C11—H11	0.9300		
			
C1—O1—C27	116.8 (3)	C15—C14—C13	117.9 (3)
C26—O4—C28	118.1 (2)	C16—C15—C14	120.8 (3)
C7—N1—C6	125.6 (2)	C16—C15—H15	119.6
C7—N1—H1N	117.2	C14—C15—H15	119.6
C6—N1—H1N	117.2	C17—C16—C15	120.3 (3)
C20—N2—C21	126.3 (3)	C17—C16—H16	119.9
C20—N2—H2N	116.9	C15—C16—H16	119.9
C21—N2—H2N	116.9	C16—C17—C18	119.3 (3)
O1—C1—C6	115.6 (3)	C16—C17—H17	120.3
O1—C1—C2	124.6 (3)	C18—C17—H17	120.3
C6—C1—C2	119.7 (3)	C17—C18—C19	121.2 (3)
C1—C2—C3	119.5 (3)	C17—C18—H18	119.4
C1—C2—H2	120.2	C19—C18—H18	119.4
C3—C2—H2	120.2	C14—C19—C18	119.4 (3)
C4—C3—C2	120.3 (3)	C14—C19—C20	123.5 (3)
C4—C3—H3	119.8	C18—C19—C20	117.1 (3)
C2—C3—H3	119.8	O3—C20—N2	124.3 (3)
C3—C4—C5	120.0 (3)	O3—C20—C19	120.0 (3)
C3—C4—H4	120.0	N2—C20—C19	115.7 (3)
C5—C4—H4	120.0	C22—C21—C26	118.6 (3)
C6—C5—C4	120.2 (3)	C22—C21—N2	125.3 (3)
C6—C5—H5	119.9	C26—C21—N2	116.1 (3)
C4—C5—H5	119.9	C21—C22—C23	120.5 (3)
C5—C6—C1	120.3 (3)	C21—C22—H22	119.7
C5—C6—N1	120.8 (3)	C23—C22—H22	119.7
C1—C6—N1	118.9 (3)	C24—C23—C22	120.5 (3)
O2—C7—N1	124.1 (3)	C24—C23—H23	119.7
O2—C7—C8	121.3 (3)	C22—C23—H23	119.7
N1—C7—C8	114.6 (2)	C23—C24—C25	119.6 (3)
C13—C8—C9	120.3 (3)	C23—C24—H24	120.2
C13—C8—C7	119.4 (2)	C25—C24—H24	120.2
C9—C8—C7	120.3 (3)	C24—C25—C26	120.1 (3)
C10—C9—C8	120.4 (3)	C24—C25—H25	120.0
C10—C9—H9	119.8	C26—C25—H25	120.0
C8—C9—H9	119.8	O4—C26—C25	124.3 (3)
C9—C10—C11	119.6 (3)	O4—C26—C21	115.1 (3)
C9—C10—H10	120.2	C25—C26—C21	120.6 (3)
C11—C10—H10	120.2	O4—C28—H28A	109.5
C12—C11—C10	120.2 (3)	O4—C28—H28B	109.5
C12—C11—H11	119.9	H28A—C28—H28B	109.5
C10—C11—H11	119.9	O4—C28—H28C	109.5
C11—C12—C13	120.6 (3)	H28A—C28—H28C	109.5
C11—C12—H12	119.7	H28B—C28—H28C	109.5
C13—C12—H12	119.7	O1—C27—H27A	109.5
C8—C13—C12	119.0 (3)	O1—C27—H27B	109.5
C8—C13—C14	121.3 (2)	H27A—C27—H27B	109.5
C12—C13—C14	119.6 (3)	O1—C27—H27C	109.5
C19—C14—C15	118.9 (3)	H27A—C27—H27C	109.5
C19—C14—C13	123.0 (3)	H27B—C27—H27C	109.5
			
C27—O1—C1—C6	169.2 (3)	C8—C13—C14—C15	−75.3 (4)
C27—O1—C1—C2	−8.3 (4)	C12—C13—C14—C15	99.9 (3)
O1—C1—C2—C3	177.3 (3)	C19—C14—C15—C16	0.3 (4)
C6—C1—C2—C3	−0.2 (4)	C13—C14—C15—C16	−175.3 (3)
C1—C2—C3—C4	−0.4 (5)	C14—C15—C16—C17	1.2 (5)
C2—C3—C4—C5	1.8 (5)	C15—C16—C17—C18	−0.8 (5)
C3—C4—C5—C6	−2.5 (4)	C16—C17—C18—C19	−1.0 (5)
C4—C5—C6—C1	1.8 (4)	C15—C14—C19—C18	−2.0 (4)
C4—C5—C6—N1	−175.7 (3)	C13—C14—C19—C18	173.3 (3)
O1—C1—C6—C5	−178.2 (3)	C15—C14—C19—C20	178.8 (3)
C2—C1—C6—C5	−0.5 (4)	C13—C14—C19—C20	−5.9 (4)
O1—C1—C6—N1	−0.6 (4)	C17—C18—C19—C14	2.4 (4)
C2—C1—C6—N1	177.1 (3)	C17—C18—C19—C20	−178.4 (3)
C7—N1—C6—C5	−65.1 (4)	C21—N2—C20—O3	−13.6 (4)
C7—N1—C6—C1	117.3 (3)	C21—N2—C20—C19	167.3 (2)
C6—N1—C7—O2	2.9 (5)	C14—C19—C20—O3	135.1 (3)
C6—N1—C7—C8	−178.7 (3)	C18—C19—C20—O3	−44.0 (4)
O2—C7—C8—C13	−50.2 (4)	C14—C19—C20—N2	−45.7 (4)
N1—C7—C8—C13	131.3 (3)	C18—C19—C20—N2	135.1 (3)
O2—C7—C8—C9	127.4 (3)	C20—N2—C21—C22	14.1 (4)
N1—C7—C8—C9	−51.1 (4)	C20—N2—C21—C26	−163.9 (2)
C13—C8—C9—C10	2.1 (5)	C26—C21—C22—C23	2.0 (5)
C7—C8—C9—C10	−175.5 (3)	N2—C21—C22—C23	−175.9 (3)
C8—C9—C10—C11	−1.6 (5)	C21—C22—C23—C24	0.9 (5)
C9—C10—C11—C12	0.0 (5)	C22—C23—C24—C25	−1.8 (6)
C10—C11—C12—C13	1.1 (5)	C23—C24—C25—C26	−0.2 (5)
C9—C8—C13—C12	−1.1 (4)	C28—O4—C26—C25	8.0 (4)
C7—C8—C13—C12	176.6 (3)	C28—O4—C26—C21	−173.0 (3)
C9—C8—C13—C14	174.1 (3)	C24—C25—C26—O4	−177.9 (3)
C7—C8—C13—C14	−8.2 (4)	C24—C25—C26—C21	3.1 (4)
C11—C12—C13—C8	−0.5 (5)	C22—C21—C26—O4	176.9 (3)
C11—C12—C13—C14	−175.8 (3)	N2—C21—C26—O4	−4.9 (3)
C8—C13—C14—C19	109.4 (3)	C22—C21—C26—C25	−4.0 (4)
C12—C13—C14—C19	−75.4 (4)	N2—C21—C26—C25	174.2 (2)

### Hydrogen-bond geometry (Å, °)

**Table:**

*D*—H···*A*	*D*—H	H···*A*	*D*···*A*	*D*—H···*A*
N1—H1N···O3^i^	0.86	2.02	2.833 (3)	157
N2—H2N···O2	0.86	2.24	3.081 (4)	167
N2—H2N···O4	0.86	2.24	2.612 (3)	106
C22—H22···O3	0.93	2.30	2.885 (4)	120

Symmetry codes: (i) *x*−1/2, −*y*+1/2, *z*−1/2.
